# Profile and quality of life of the adult population in good health according to the level of vitality: European NHWS cross sectional analysis

**DOI:** 10.1186/s12889-023-15754-0

**Published:** 2023-06-05

**Authors:** Anne-Laure Tardy, Sophie Marguet, Halley Costantino, Andrew Stewart, deMauri Mackie, Grèce Saba, Caroline Amand

**Affiliations:** 1 Science Hub, Sanofi Consumer Healthcare, Gentilly, France; 2 Cerner Enviza, Paris, France; 3 Cerner Enviza, Malvern, PA USA; 4 Science Hub, Sanofi Consumer Healthcare Cambridge, Cambridge, MA USA

**Keywords:** Vitality, Fatigue, Good health, NHWS, Patient profile, Patient activation, Health-related quality of life

## Abstract

**Background:**

The World Health Organization’s definition of health highlights the importance of mental and physical wellbeing and not only disease state. However, lack of awareness on the burden of impaired vitality and its impact on the quality of life of the general healthy population prevents healthcare providers from delivering appropriate solutions and advice. This study aims to better characterize this population in Europe and identify the profile and the health reported outcomes associated with impaired vitality.

**Methods:**

This retrospective observational study included National Health and Wellness Survey (NHWS) data collected in healthy participants aged 18–65 years from five European Union countries in 2018. Socio-demographic and lifestyle characteristics, comorbidities, attitudes towards healthcare systems, Patient Activation Measure, health-related quality of life outcomes (EQ-5D), and work productivity and activity impairment were analysed according to SF-12 vitality score subgroups (≥ 60, 50– < 60, 40– < 50, < 40).

**Results:**

A total of 24,295 participants were enrolled in the main analysis. Being a female, younger, having a lower income and being obese or having sleep and mental disorders was associated with an increased risk of impaired vitality. This was associated with a higher consumption of healthcare resources along with having a weak patient-physician relationship. Participants who were disengaged in the self-management of their health were 2.6 times more likely to have a low level of vitality. For participants in the lowest vitality group, odds of mobility problems increased by 3.4, impairment of usual activity by 5.8, increased of pain and discomfort by 5.6 and depression and anxiety by 10.3, compared with participants in the highest vitality group. Also, odds of presenteeism increased by 3.7, overall work impairment by 3.4 and daily activity losses by 7.1.

**Conclusion:**

Evidence-based trends facilitate the identification of a healthy population with impaired vitality in real-world practice. This study highlights the actual burden of low vitality on daily life activities, particularly on mental health and reduced work productivity. Additionally, our results underline the importance of self-engagement in the management of vitality impairment and highlights the need to implement strategies to address this public health concern in the affected population (HCP-patient communication, supplements, meditation).

**Supplementary Information:**

The online version contains supplementary material available at 10.1186/s12889-023-15754-0.

## Background

The World Health Organization’s definition of health highlights the importance of mental, physical and social well-being in everyday life. Fatigue, best viewed as a continuum [1], is a multidimensional concept that is often described subjectively as a lack of energy, vitality, and motivation [1, 2] occurring at differing levels of severity [3]. In the general population, the prevalence of fatigue is broad, ranging from 15 to 37.9% depending on the definition applied, the tool used for assessment, the data source and the country [3–7]. In fact, one of the critical challenges faced when designing studies in this area is the high number of tools for reporting fatigue, and the absence of clear cut-off values for an accurate definition [8, 9].

Fatigue is a well-known, common and debilitating symptom associated with many different illnesses, including cancer [10–14], inflammatory bowel diseases [15–17], rheumatic diseases [18, 19], Parkinson's disease [20], liver disease [21, 22], type 1 diabetes [23] and multiple sclerosis [24]. Nevertheless, based on data obtained in primary care, a considerable proportion of self-reported fatigue cannot be explained by any underlying disease [25–27]. Studies focused on describing fatigue in the general population have shown that increased levels of fatigue are associated with socio-demographic components (e.g., female sex [3–7, 28, 29], younger age [5, 7, 28], lower level of education [6, 7, 29] and low income [6, 28]); medical conditions (e.g., obesity [3, 5], anxiety/depression [4, 5, 7], stress [4], sleep disorders [4, 5, 7], number of health conditions [6, 7, 29]); medication intake (e.g., analgesics [3], antidepressants [5]), lifestyle factors (e.g., low level of physical activity [3, 28], unbalanced diet [3, 30]); and with an overall decreased self-reported quality of life [6]. In addition, fatigue was found to impact welfare and result in substantial economic costs (e.g., decline of work and household productivity, increased level of absenteeism, increased healthcare consumption) [6, 31–33]. Large multi-centric studies addressing these factors in a population in good health are necessary to aid the identification of the population at risk of fatigue by healthcare professionals and patients [31–33].

Additionally, patient and healthcare professionals have limited awareness of the burden of low vitality on quality of life. This was found to negatively impact patient-doctor relationships (e.g., longer visits, bad communication and co-operation, decreased empathy, reduced confidence, perception of complaint seriousness) and may prevent appropriate recommendations and solutions [34–36]. This is of major public health interest and an improvement in the ability to evaluate fatigue and its burden is required.

The National Health and Wellness Survey (NHWS) is an internet-based questionnaire self-administered to a sample of adults aged ≥ 18 years in several countries globally (US, France, Germany, Italy, Spain, UK, Japan, China, South Korea, Taiwan, Brazil, and Russia). It is completed by all participants in real-world settings and consists of a base survey component used to assess demographics, diseases experienced and diagnosed, and outcomes like health-related quality of life, work productivity and activity impairment. More interestingly, it captures vitality in the general population through the widely used short form-12 (SF-12) questionnaire [37–39]. The survey is completed by a large number of participants, offering the opportunity to conduct different types of studies [40–43] and to focus on a selected population in good health. Existing self-administered surveys targeting the population in good health are rare, therefore this database is of major interest for our research question.

The primary objective of this study was to describe the characteristics of a pooled European adult population in good health according to the level of vitality in the NHWS database. The secondary objectives were to identify the specific profile associated with the different levels of vitality and to describe participant health reported outcomes according to their level of vitality.

## Methods

### Study design and data source

This study was a population-based, retrospective observational study, which included NHWS data collected from participants in five European countries (France, Germany, Italy, Spain, and the United Kingdom [UK]) from April to July 2018. Due to the potential impact of the coronavirus disease-19 (COVID 19) pandemic on fatigue and impaired vitality, the data obtained in 2019 and 2020 were not considered for our study.

**Fig. 1:**
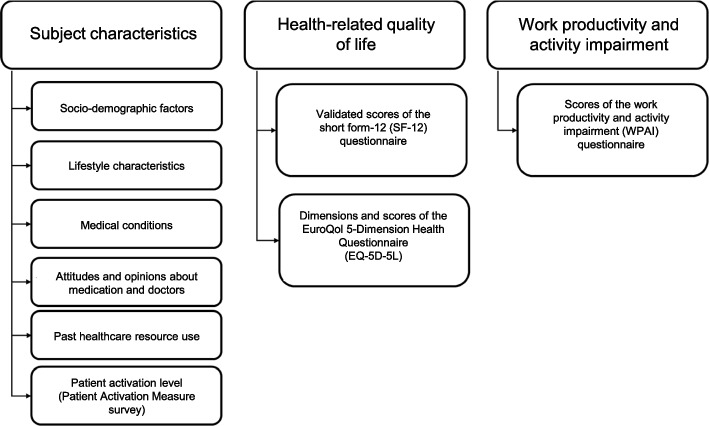
National Health and Wellness Survey modules EQ-5D-5L, EuroQol 5-Dimension Health Questionnaire; NHWS, National Health and Wellness Survey; SF-12, short form-12; WPAI, Work Productivity and Activity Impairment

The NHWS aims to reflect the general population of the country surveyed using known population incidences for key demographic characteristics. Potential participants are recruited through an existing, general-purpose web-based consumer panel that is maintained by Lightspeed Research and its partners, with quota sampling within the survey panel to ensure representativeness in terms of age and gender. The consumer panel recruits its members through opt-in emails, co-registration with panel partners, e-newsletter campaigns, banner placements, and affiliate networks. The different components of the survey are described in Fig. 1.

The NHWS was validated for exemption determination by the Pearl Institutional Review Board (IRB) in February 2018.

### Analyzed population

Participants were eligible if they responded to the NHWS survey held in 2018 in France, Germany, Italy, Spain or the UK. Other inclusion criteria were age (18 to 64 years) and being in “good health”. Respondents older than 65 years were not retained for the main analysis due to the bias induced by the criteria for good health.

As detailed in Additional file 1, the population in good health was defined using an iterative approach by a rigorous revision of around 130 reported medical conditions. The assessment considered the chronicity, severity or the combination of medical conditions, as well as the current utilization of prescription medicine. Patients self-reporting dementia were also excluded due to the potential untrusted nature of their answers.

Vitality score was collected using one of the twelve items of the SF-12 questionnaire. It is standardized on a 0 to 100 metric, with higher scores representing greater vitality level. Participants were stratified into four subgroups of vitality score < 40 (lowest vitality), 40– < 50, 50– < 60, ≥ 60 (highest vitality) based on the distribution of the score in the analyzed population.

The five European countries were considered homogenous enough in terms of vitality to be pooled as the difference of mean vitality score between countries for the overall population and the population in good health was < 5 (minimally clinically important difference) [44]. Country was nonetheless kept as a covariate of adjustment to avoid bias.

### Outcomes

#### To define participant profile according to vitality score

Data on demographics, lifestyle characteristics, medical conditions, attitudes towards doctors, past healthcare resource use and patient activation measure (PAM®) levels were gathered at the time of the survey. All variables were reported for the total population and according to the four previously established vitality groups (< 40, 40– < 50, 50– < 60, ≥ 60).

Demographic variables collected included age (years), gender, marital status, education, employment status and household income in the past year. For lifestyle characteristics, data on smoking status, alcohol use, exercise in the past month and body mass index (BMI) were reported. Medical conditions that occurred at any time, or in the past 12 months from a predefined list of around 130 were considered (Additional file 1). Participant opinions toward doctor attention to needs and concerns were also evaluated, and answers were rated using a 5-point scale (“strongly disagree”, “disagree”, “neither disagree nor agree”, “agree”, or “strongly agree”). Self-reported healthcare resources used in the 6 months prior to the survey were collected and included emergency room visits, hospitalizations and visits to either a general practitioner (GP), psychologist or therapist, or any other healthcare specialists.

The 13-item PAM assessment segments individuals into one of four activation levels along a 100-point empirically derived continuum [45, 46]. Patient activation refers to an individual's knowledge, skill, and confidence in managing one’s own health. More activated patients (PAM level 4) are more likely to adhere to treatment regimens, engage in disease-specific self-management behaviours, and have better health outcomes compared with the lowest PAM level (level 1) [47].

#### To evaluate the burden of impaired vitality on participant daily life

Data regarding quality of life (SF-12 and EuroQol 5-Dimension Health Questionnaire [EQ-5D-5L]) and productivity (Work Productivity and Activity Impairment [WPAI]) were collected by questionnaire at the time of the survey. All variables were reported for the total population and according to the four previously established vitality groups (< 40, 40– < 50, 50– < 60, ≥ 60).

The seven remaining sub-scores of the SF-12 (physical functioning, physical role functioning, bodily pain, general health, social functioning, emotional role functioning and mental health), along with the physical component summary (PCS) and the mental component summary (MCS) are standardized on a 0 to 100 metric, with higher scores representing better health status.

EQ-5D-5L is a self-reported questionnaire consisting of five dimensions (mobility, self-care, usual activities, pain/discomfort, and anxiety/depression), to which participants respond with their current level of problems according to a 5-point scale (none, slight, moderate, severe, or extreme). Responses are used to derive the EQ-5D-5L Index Score. Lower overall index scores indicate higher disability [48], with a score of 1 corresponding to a perfect health state and 0 corresponding to death. In addition, the EQ-5D- 5L visual analogue scale (VAS) allowed participants to score their health of the day from 0 (worst health you can imagine) to 100 (best health you can imagine) [48].

WPAI Questionnaire is a six-item validated measure, which consists of four metrics: absenteeism (% of work time missed due to health in last seven days), presenteeism (% of impairment experienced at work due to health in the last seven days), overall work productivity loss (an overall impairment estimate that is a combination of absenteeism and presenteeism) and activity impairment (% of impairment in daily activities due to health in last seven days) [49]. Only participants who reported being full-time, part-time, or self-employed provided data for absenteeism, presenteeism, and overall work impairment. All participants provided data for activity impairment.

### Statistics

Socio-demographic and lifestyle characteristics, medical conditions, past healthcare resource use and PAM were described using common descriptive statistics and compared using chi-square tests and analysis of variance (ANOVA) according to the level of vitality. To identify the specific patient profile associated with the different levels of vitality, a multivariate ordinal logistic regression model was conducted to assess their relationship. Odds ratio (OR) and 95% confidence intervals (CI) were provided.

To assess the relationship with quality of life, SF-12 (MCS, PCS and the 7 other domains scores), EQ-5D (5 dimensions, EQ-5D-5L index score and VAS) and WPAI (4 metric scores) were described using common descriptive statistics and compared using chi-square tests and ANOVA. Multivariate analyses were also conducted using logistic regression models for each dimension of the EQ-5D-5L (any problems vs no problem), absenteeism (> 0% vs 0%), presenteeism (> 0% vs 0%), overall work (> 0% vs 0%), and activity impairment (> 0% vs 0%). The vitality score in four groups was added as a covariate and other covariates were selected based on the results of descriptive and bivariate analyses, with OR and 95% CI provided.

## Results

### Analyzed population

In total, 24,295 adults in good health aged between 18 and 64 years old were selected (participants). They were then assigned to one of four vitality groups based on the statistical distribution of the vitality score of the SF-12 questionnaire: < 40: lowest vitality (*N* = 4,173), 40– < 50 (*N* = 9,327), 50– < 60 (*N* = 9,059) ≥ 60: highest vitality (*N* = 1,736).

### Population profile according to vitality score

#### Socio-demographics and lifestyle characteristics

For the analysis population, the mean age was 40.3 (Standard deviation [SD] 12.7) years and 54.6% of participants were female. The majority of participants were married or living with a partner (56.2%), currently employed full time (53.1%) and presented with a level of education lower than college (52.0%) (Additional file 2).

There were significant differences in age, gender, marital status, household income, employment status, exercise, BMI, and smoking status across the vitality groups. Participants in the highest vitality group were slightly older than those in the lowest vitality group (41.2 years versus 38.2 years). Additionally, there were more women (61.8 vs 43.3%), single people (50.0 vs 38.2%), unemployed (29.9 vs 19.2%), sedentary (43.9 vs 24.2%), obese persons (15.4 vs 8.5%) and fewer non-smokers (53.9 vs 61.1%) in the lowest vitality group compared with the highest. No significant differences were noted among vitality groups for the level of education or alcohol use (Additional file 2).

#### Medical condition and comorbidities

The mean number of comorbidities reported as diagnosed by a physician was 1.3 (SD 1.8) per participant in the analysis population, and the level of each comorbidity encountered was always below 10.0%. The most common comorbidities were allergies (9.0%), pain (7.7%), hay fever (7.6%), depression (7.4%), anxiety (6.1%), high blood pressure/ hypertension (6.0%) and high cholesterol (5.4%) (Additional file 3).

The participants in the lowest vitality group reported a greater mean number of comorbidities (1.8 vs 0.6) compared with participants in the highest vitality score group. The prevalence of the most common comorbidities was significantly different across groups. Depression (15.7 vs 2.0%), anxiety (11.8 vs 2.0%) and high blood pressure (7.3 vs 3.5%) had the greatest differences between groups, with higher prevalence in the lowest vitality group compared with the highest, respectively (Additional file 3).

#### Attitudes and opinions about medication and doctors

In the lowest vitality score group, 40% of the participants “strongly agreed” or “agreed” that “their doctor is attentive to their needs” compared with 53.9% in the highest group. Furthermore, the ‘strongly agreed’ response was only recorded by 8.6% of participants in the lowest vitality group compared to 24.3% in the highest group.

#### Healthcare consumption in the past six months

Several trends were observed among the population that experienced at least one event of healthcare resource utilization in the last 6 months. In particular, the lowest level of vitality group was associated with a higher number of participants that visited GPs (51.2 vs 35.3%) and psychologists/therapists (3.4 vs 0.6%) in comparison with the highest vitality score group.

#### Patient activation measure levels

**Fig. 2:**
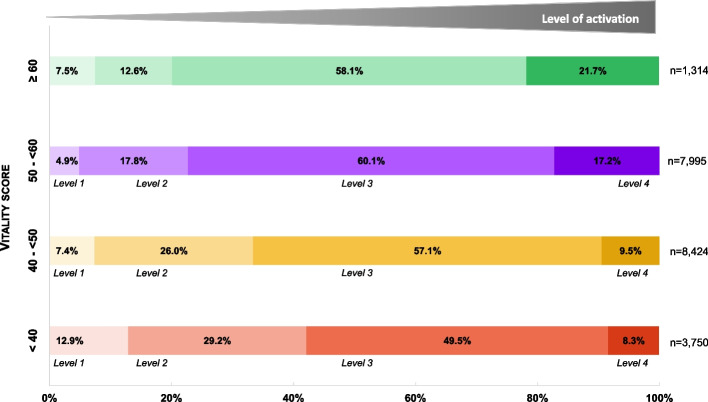
Patient activation measure according to vitality groups Percentages were calculated on respondents (*N* = 21,483)

In the analysis population, more than half of the participants had a level of activation of 3 in the PAM, corresponding to individuals who have the key facts and can act and take control of their health (Fig. 2). However, the lowest vitality group contained more participants who had a lack of knowledge and confidence for managing their own health, compared with participants in the highest group (sum of PAM level 1 and 2: 42.1 vs 20.1%) (Fig. 2).

#### Summary of participant profile according to vitality score

**Fig. 3:**
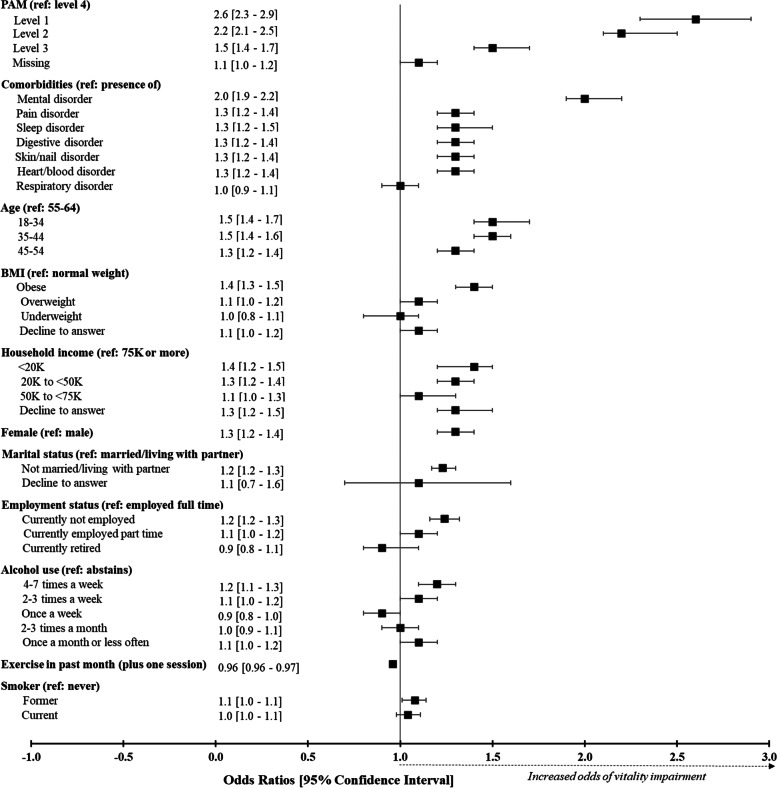
Multivariate analysis of the association between the characteristics of the population (participant profile) and the vitality groups. BMI, body mass index; PAM, patient activation measure **a** mental disorders: Anxiety (no current use of medications), depression (no current use of medications or not severe when taking medications), others without current use of medications (attention deficit disorder, attention deficit hyperactivity disorder, bipolar disorder, generalized anxiety disorder, obsessive compulsive disorder, panic disorder, phobias, post-traumatic disorder, social anxiety disorder). **b** pain disorders: Headache (no current use of medications), migraine (no current use of medications), pain (no current use of medications or not severe when taking medications). **c** sleep disorders: Insomnia (no current use of medications or not moderate to severe when taking medications), others (narcolepsy (no current use of medications), sleep apnea (not severe when taking medications), other sleep difficulties (no current use of medications)). **d** digestive disorders: GERD / acid reflux, heartburn, others (chronic constipation, diarrhea (frequent), diverticulitis, ulcers (active/peptic stomach or duodenal, no current use of medications)). **e** skin/ nail disorders: Acne, dermatitis (no current use of medications or not moderate to severe when taking medications), eczema (no current use of medications or not moderate to severe when taking medications), others (Atopic dermatitis (not moderate to severe when taking medications), Fungal infections of the skin or Athlete’s foot, hidradenitis suppurativa, rosacea, shingles, skin ulcers/cellulitis). **f** heart/blood disorders: Type 2 Diabetes (T2D) (not associated with high blood pressure or high cholesterol if current use of medications for these conditions), High blood pressure (not associated with T2D or high cholesterol if current use of medications for these conditions), High cholesterol (not associated with T2D or high blood pressure if current use of medications for these conditions). **g** respiratory disorders: Allergies (no current use of medications), asthma (no current use of medications), hay fever

Most of the characteristics identified as significantly different between the vitality groups in the bivariate analyses remained significant in the multivariate analysis (Fig. 3). The following socio-demographic characteristics significantly increased the odds of vitality impairment: being female (OR: 1.3 [95%CI:1.2–1.4]); being younger (18–34 years old vs 55–64 years old, OR: 1.5 [95%CI 1.4–1.7]); being single (OR: 1.2 [95%CI 1.2–1.3]); having a lower annual income (< 20,000 vs > 75,000 euros, OR: 1.4 [95%CI 1.2–1.5]); and being currently unemployed (OR 1.2 [95%CI 1.2–1.3]). Being obese (OR 1.4 [95%CI 1.3–1.5]) and having exercised less in the past month (OR 1.0 [95%CI 1.0–1.0]) significantly increased the odds of vitality impairment as well.

With an OR of 2.6 (95%CI 2.3 – 2.9), PAM level 1 (lack of knowledge and confidence for managing their own health) was the characteristic most associated with vitality impairment. “Mental disorder” (anxiety, depression, and others) (OR 2.0 [95%CI 1.9 – 2.2]), “sleep disorders” (OR:1.3, [95%CI 1.2–1.5]) and “pain disorders” [OR: 1.3, 95%CI 1.2–1.4] were also medical conditions that significantly increased the odds of vitality impairment in the population in good health.

### Health-Related Quality of Life outcomes according to vitality score

#### SF-12 scores

**Fig. 4:**
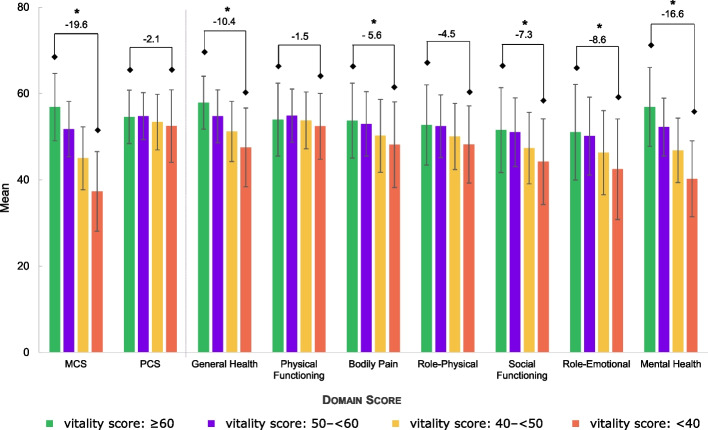
SF-12 scores by domains according to vitality groups MCS, mental component summary; PCS, physical component summary; SD, standard deviation; SF-12, short form-12. * Clinically meaningful of at least 5 points of incremental difference between higher vitality score group (≥ 60) and lowest vitality score group (< 40); Error bars show SD. All participant of the main analysis completed the SF-12 questionnaire (*N* = 24,295). Number of participants by group was as follows: > 60: *N* = 1,736; 50—< 60: *N* = 9,059; 40—< 50: *N* = 9,327; < 40: *N* = 4,173

The participants in the lowest vitality group reported lower mean MCS and PCS compared with participants in the highest vitality score group. In addition, the mean scores of all seven domains of the SF-12 significantly decreased together with the vitality score. Considering that at least 5 points is a clinically meaningful difference, all the domains except the physical functioning and the role-physical scores showed a meaningful difference between the highest and lowest vitality groups (Fig. 4).

#### EuroQol 5-dimension health questionnaire

**Fig. 5:**
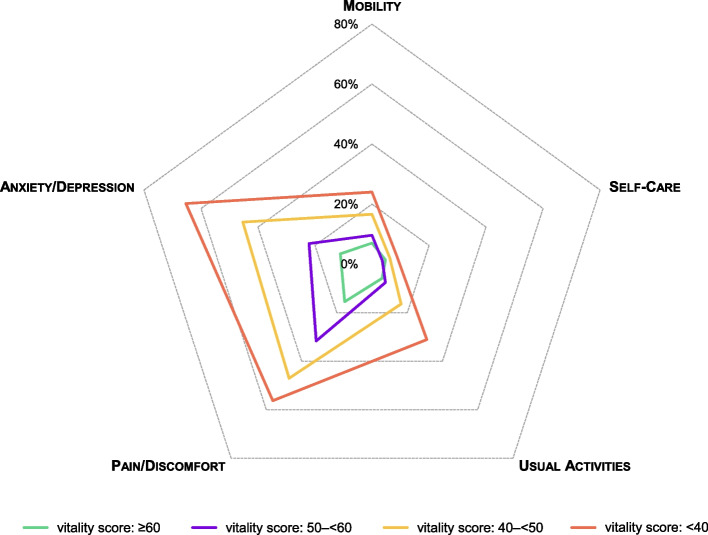
Proportion of participants reporting problems in EQ-5D-5L questionnaire according to vitality groups EQ-5D-5L, EuroQol 5-Dimension Health. Number of participants by group was as follows: ≥ 60: *N* = 1,736; 50– < 60: *N* = 9,059; 40– < 50: *N* = 9,327; < 40: *N* = 4,173

The high mean EQ-5D-5L utility index of 0.9 (SD 0.1) and mean VAS of 80.0 (SD 18.6) were consistent with a population in good health (Fig. 5, Additional file 4). However, the scores decreased corresponding with a decrease in the level of vitality across the groups. For the five dimensions of the EQ-5D-5L, a large majority of participants reported having no problems with mobility (85.4%), self-care (94.4%), usual activities (85.1%), pain/discomfort (59.3%) and anxiety/depression (62.3%), confirming that the selected population was in good health.

When considering the participants reporting at least one problem, those in the lowest vitality group were significantly more concerned by pain/discomfort (56.3 vs 15.5%) and by anxiety/depression (65.3 vs 11.2%) compared with the highest vitality score group.

#### Work productivity and activity impairment over past seven days

**Fig. 6:**
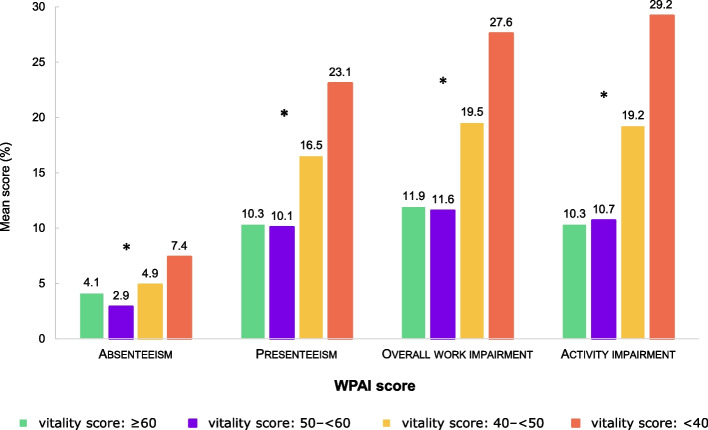
Work productivity and activity impairment (WPAI scale components) according to vitality groups WPAI, Work Productivity and Activity Impairment. * Significantly different between 4 groups (*p* < 0.01). Active population (*N* = 16,788) completed the absenteeism and overall work impairment questions. Active population who declared working at least 1 h during the 7 last days (*N* = 16,639) completed the question about presenteeism. All participant (*N* = 24,295) completed the question related to activity impairment

Overall, 16,788 participants completed the productivity assessments due to their active employment status, including 16,639 participants who declared working at least 1 h during the past 7 days and answered the question related to the presenteeism, while all 24,295 participants in the analysis population completed the question related to daily activity. The selected population who completed the WPAI reported levels on work absenteeism (4.4%), presenteeism (14.5%), overall work impairment (17.8%) and daily activity impairment (17.1%). The low level of absenteeism confirmed the good health profile of our total population; however, there were significant (*p* < 0.001) differences for each of the 4 WPAI parameters between the vitality groups. Results between the lowest and highest impaired vitality groups on absenteeism (7.4 vs 4.1%), presenteeism (23.1 vs 10.3%), work impairment (27.6 vs 11.9%) and activity impairment (29.2 vs 10.3%) scores confirmed the true impact of impaired vitality on all productivity parameters (Fig. 6).

#### Summary of the burden of impaired vitality on participant daily life

**Fig. 7:**
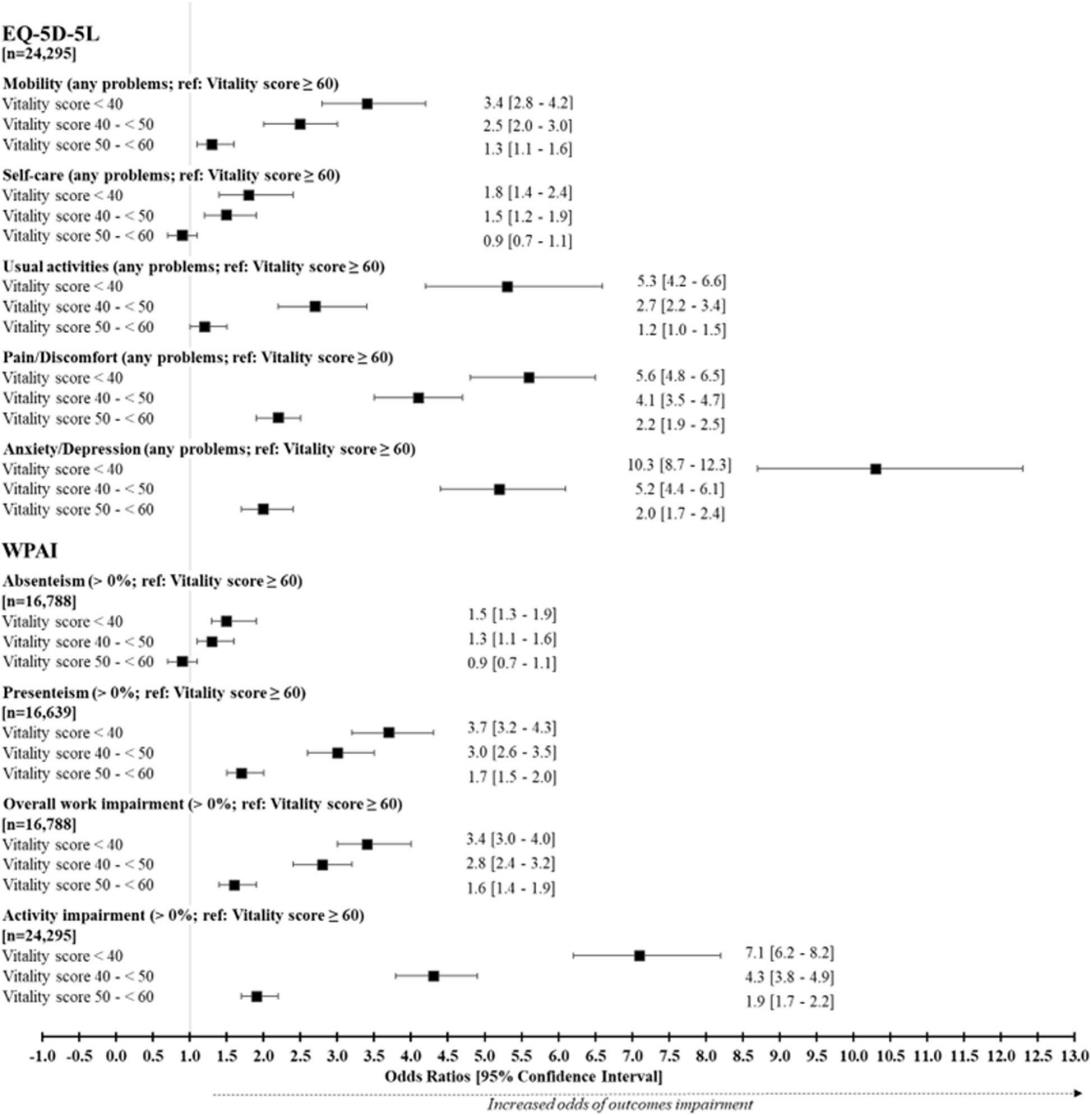
Multivariate analyses of the association between the health outcomes and the vitality groups EQ-5D-5L, EuroQol 5-Dimension Health Questionnaire; OR, odds ratio; WPAI, work productivity and activity impairment

The significant relationships between impaired vitality and Health-Related Quality of Life outcomes were confirmed using a multivariate analysis, adjusted by profile parameters. The highest vitality group was used as a reference, with the results for each outcome presented in Fig. 7.

Between the highest and lowest vitality groups, the odds showed an increased risk of reporting any problems for all dimensions of the EQ-5D-5L questionnaire (OR [95% CI]) for < 40 vs ≥ 60: mobility 3.4 [2.8 – 4.2], self-care 1.8 [1.4 – 2.4], usual activities 5.3 [4.2 – 6.6] and pain/discomfort 5.6 [4.8 – 6.5], as well as increased concern of anxiety/depression 10.3 [8.7 – 12.3]. Lastly in the WPAI, the odds showed a significant increase in the risk of work impairment, especially presenteeism (OR: 3.7 [95%CI 3.2 – 4.3]) and activity impairment (OR: 7.1 [95%CI 6.2 – 8.2]) between the highest and lowest vitality groups.

## Discussion

It is our understanding that the present study, which involved 24,295 healthy adults across five European countries, is the largest performed in this under-investigated area of the association between mental and physical wellness, vitality, and fatigue. Data related to patient profile, healthcare habits, empowerment, and health economic burden of fatigue in the same sample of participants was assessed. As a result, this study reinforces previous data obtained from smaller, monocentric studies regarding patient socio-demographic attributes, lifestyle and health condition characteristics and healthcare consumption. As previously reported, we contribute to existing evidence that being female, younger and having a lower income was associated with an increased risk of feeling fatigued, even in the population in good health [3–7, 28, 29]. Increased sedentary behaviors were also associated with a higher feeling of fatigue [3, 28]; however, contrary to previous knowledge [6, 7, 29], level of education had no impact on the feeling of fatigue. In line with previous studies, we demonstrated that a number of health conditions, particularly obesity, sleep and mental disorders, had a high impact on the reported vitality level [3–7, 29]. Additionally, we showed that an impaired vitality level was associated with an increased consumption of healthcare resources and GP visits. The latter was associated with a weak patient-GP relationship, with only 8.6% of participants in the lowest vitality group strongly agreeing that their doctor is attentive to their needs [34, 35, 47, 50–52]. Improved characterization of the patient profile could therefore better aid healthcare providers to identify the population suffering from vitality impairment in real-world settings.

Furthermore, for the first time we established the importance of patient engagement on the level of vitality. Our data showed that healthy patients that were disengaged and overwhelmed by the self-management of their health (PAM level 1) were 2.6 times more likely to have a low level of vitality, suggesting that self-empowerment is a key actionable factor for preventing vitality impairment and its associated health consequences. These established links between engagement and better health and economic outcomes are of major public health importance [52–55]. Further analysis should be conducted to evaluate the beneficial impact of patient engagement on health and the economy in this specific healthy population with impaired vitality level.

Finally, this study demonstrated the burden of impaired vitality on the quality of life and work productivity of a healthy population. For participants in the lowest vitality group, odds of mobility problems increased by 3.4, impairment of usual activity by 5.8, pain and discomfort by 5.6 and depression and anxiety by 10.3, compared with participants in the highest vitality group. Odds of presenteeism also increased by 3.7, overall work impairment by 3.4 and daily activity losses by 7.1. These results are consistent with similar data obtained in a general population of workers who missed an average of 4.1 (95% CI: 3.8–4.4) productive work hours per week due to fatigue [6]. The meaningful impact of vitality impairment on the population in good health provides evidence that public health initiatives are required to better identify and consider this population as impaired.

A strength of the NHWS database is that it allows for the selection of a population in good health. The EQ-5D-5L index of our selected population (0.89) confirmed that our definition of good health and the individual’s self-reported good health status was reliable compared with those not retained in our analysis (0.78). A feasibility analysis confirmed that data from all five European countries could be pooled for analysis without introducing bias due to the subjective nature and potential cultural impact; however, further analysis should be conducted by splitting by country to further evaluate the local/country influence (e.g., the economic impact of the burden of low vitality).

This study had several limitations, one of which is the use of the vitality sub-item of the SF-12 questionnaire to determine vitality, as it is not validated for fatigue assessment [9]. However, there are extensive examples in research and real-world settings in which fatigue and vitality were not captured using a validated tool [6], including sub-items of the SF-36 [56–58] and SF-12 [59, 60] questionnaires. In addition, accessible approaches to evaluate fatigue in real practice are required, and the instrumental use of SF-12 score to represent the severity spectrum of self-experienced vitality could be a useful and simple tool. Further investigations should include validation of the SF-12 sub-item to confirm its ability to detect the low vitality threshold in general practice. Moreover, results obtained for the MCS and PCS of the SF-12 questionnaire need to be interpreted with caution as the vitality domain score is used in both component summary scores, with a higher weight for the MCS [61]. Finally, a lower number of participants in the highest vitality score group responded to the PAM survey, which is consistent with a population in better health.

The present study confirms that even low to moderate sleep disorders, stress and anxiety had a high negative impact on the vitality level of a healthy population. In the meantime, there is increasing evidence of the psychological impact of the COVID-19 pandemic [62, 63], particularly on sleep disorders [64, 65] and on the level of stress and anxiety [66, 67]. Therefore, determining the impact of the COVID-19 pandemic on the population in good health would be insightful, and could be achieved by analysing the 2020 NHWS data in the European Union. This analysis would, for instance, assess the prevalence of impaired vitality level, patient engagement in their health and burden during the pandemic, and the subsequent impact on work productivity and quality of life compared with pre-pandemic data.

## Conclusion

The trends observed can be used to profile the healthy population with impaired vitality (being a woman, younger, single, obese, presenting with mild mental disorders and having a low income) and may facilitate the identification of this population in real-world practice. This study established the actual burden of low vitality on daily life activities, particularly on the risk of anxiety and depression and reduced work productivity. Also, our data suggest that the population presenting with impaired vitality may reduce health consequences and daily life burden due to low vitality by being more engaged in the management of their health. This key factor is of high importance for establishing new and innovative health strategies to modify the behaviour among the population who are unaware of the burden of low vitality. Propositions should include tools for better interaction with healthcare providers on this specific topic since this population does not feel doctors are attentive to their needs. Other approaches could include the use of vitamins and minerals supplements [30, 68, 69], which are commonly used for fatigue management [70], cognitive and behavioural interventions [71], and sleep routine or meditation [72]. Finally, a major step forward in this area would be developing specific tools to measure the impact on vitality, as well as the validation of the vitality score sub-item of the SF-12 questionnaire to assess fatigue and vitality, which would provide a simple and reliable tool for common use.

## Supplementary Information

**Additional file 1. **Overview of the medical conditions explored in the NHWS study and their exclusion or partial exclusion for the present retrospective study.

**Additional file 2. **Sociodemographic and lifestyle characteristics: Total healthy population and according to vitality scores.

**Additional file 3. **Medical conditions and comorbidities: Total healthy population and according to vitality scores.

**Additional file 4. **Descriptive statistics of the EQ-5D-5L according to the vitality score.

## Data Availability

The data that support the findings of this study are available from Cerner Enviza but restrictions apply to the availability of these data, which were used under license for the current study, and so are not publicly available. Data are however available from the authors upon reasonable request and with permission of Cerner Enviza.

## Abbreviations

ANOVA: Analysis of variance
BMI: Body mass index
CI: Confidence interval
COVID 19: Coronavirus disease-19;
EQ-5D-5L: EuroQol 5-Dimension Health Questionnaire
GP: General practitioner
IRB: Institutional Review Board
MCS: Mental component summary
NHWS: National Health and Wellness Survey
OR: Odds ratio
PAM: Patient activation measure
PCS: Physical component summary
SD: Standard deviation
SF-12: Short form-12
VAS: Visual analogue scale
WPAI: Work Productivity and Activity Impairment
