# Supplementary material for: Profile and quality of life of the adult population in good health according to the level of vitality: European NHWS cross sectional analysis
Source: BMC Public Health. 2023 Jun 5;23:1061. doi: 10.1186/s12889-023-15754-0 (PMC10239722; doi:10.1186/s12889-023-15754-0)
Supplement: Supplementary file 1 — Additional file 1. Overview of the medical conditions explored in the NHWS study and their exclusion or partial exclusion for the present retrospective study. [file 12889_2023_15754_MOESM1_ESM.docx]

Additional file 1. Medical conditions explored in the NHWS study and their exclusion from the present retrospective study

| **Following chronic diseases were excluded from the definition of the population in good health** | |
| --- | --- |
| Cancer |  |
| Digestive diseases: | Crohn’s disease, Irritable bowel syndrome, Ulcerative Colitis |
| Mental diseases: | Schizophrenia |
| Heart or blood diseases: | Angina, Arrhythmia, Atherosclerosis, Atrial fibrillation, Congestive Heart Failure, Deep vein thrombosis, Type 1 Diabetes, Latent Autoimmune Diabetes, Gestational diabetes, Heart attack, Heart murmur, Hemophilia A, Hemophilia B, Left ventricular hypertrophy, Mini stroke / Transient Ischemia Attack, Peripheral arterial disease, Peripheral vascular disease, Pulmonary embolism, Sickle cell disease, Stroke, Unstable Angina / Chest pains |
| Infectious diseases: | AIDS / HIV, Hepatitis B, Hepatitis C |
| Inflammation / Immunology diseases: | Ankylosing spondylitis, Lupus, Psoriatic Arthritis, Rheumatoid Arthritis |
| Liver diseases: | Chronic liver disease, Cirrhosis, Non-Alcoholic Fatty Liver Disease, Non-alcoholic Steatohepatitis |
| Neurological diseases: | Dementia, Hemiplegia, Multiple sclerosis, Muscular dystrophy, Parkinson’s disease |
| Pain: | Fibromyalgia, Diabetic neuropathic pain |
| Respiratory diseases: | Chronic cough, Chronic obstructive pulmonary disease, Chronic bronchitis, Emphysema, Cystic fibrosis |
| Skin or nail diseases: | Chronic hives, Psoriasis |
| Urologic or kidney diseases: | Benign prostatic hyperplasia, Chronic kidney disease, Moderate or severe renal / kidney disease, Overactive bladder (dry / wet), Stress urinary incontinence |
| Women’s health diseases: | Endometriosis |
| **Medical conditions excluded when the participants reported currently using a prescription medication**  As this is a cross-sectional study, it has been considered that the current use of a prescription medication was not consistent with a population in good health for the following medical conditions | |
| Ulcers, Anxiety, Attention deficit disorder, Attention deficit hyperactivity disorder, Bipolar disorder, Generalized anxiety disorder, Obsessive compulsive disorder, Panic disorder, Phobias, Post-traumatic stress disorder, Social anxiety disorder, Erectile dysfunction, Epilepsy, Restless legs syndrome / Willis Ekbom disease, Headache, Migraine, Allergies Asthma, Sleep difficulties (other than insomnia, narcolepsy, or sleep apnea), Narcolepsy, Fibroids | |
| **Participants were also excluded if they reported currently using a prescription medication for at least two of the following medical conditions** | |
| Type 2 Diabetes, High blood pressure (hypertension), High cholesterol | |
| **The following medical conditions were excluded if the disease was considered too severe when taking a medication to be consistent with a population in good health** | |
| Depression (exclusion of severe patients), Osteoarthritis (exclusion of severe patients), Osteoporosis (exclusion of severe patients), Pain (exclusion of severe patients), Atopic dermatitis (exclusion of moderate and severe patients), Dermatitis (exclusion of moderate and severe patients), Eczema (exclusion of moderate and severe patients), Insomnia (exclusion of moderate and severe patients), Sleep apnea (exclusion of severe patients) | |
|  | |
