# Supplementary material for: Profile and quality of life of the adult population in good health according to the level of vitality: European NHWS cross sectional analysis
Source: BMC Public Health. 2023 Jun 5;23:1061. doi: 10.1186/s12889-023-15754-0 (PMC10239722; doi:10.1186/s12889-023-15754-0)
Supplement: Supplementary file 2 — Additional file 2. Sociodemographic and lifestyle characteristics: Total healthy population and according to vitality scores. [file 12889_2023_15754_MOESM2_ESM.docx]

Additional file 2. Sociodemographic and lifestyle characteristics: Total healthy population and according to vitality scores

| **Characteristics** | | **Total population**  **(N=24,295)** | **Vitality score** | | | | | | **P value** |
| --- | --- | --- | --- | --- | --- | --- | --- | --- | --- |
|  |  |  | **< 40  (N=4,173)** | **40–<50 (N=9,327)** | **50–<60  (N=9,059)** | **≥60  (N=1,736)** | |  | |
| **Age in years** | Mean (SD) | 40.3 (12.7) | 38.2 (12.6) | 39.6 (12.5) | 41.7 (12.7) | 41.2 (13.0) | **<0.001^a^** | | |
| **Age in years, n (%)** | 18–34 | 9,000 (37.0%) | 1,835 (44%) | 3,583 (38.4%) | 2,982 (32.9%) | 600 (34.6%) | **<0.001^a^** | | |
|  | 35–44 | 5,780 (23.8%) | 966 (23.1%) | 2,293 (24.6%) | 2,097 (23.1%) | 424 (24.4%) |  | | |
|  | 45–54 | 5,352 (22.0%) | 801 (19.2%) | 2,034 (21.8%) | 2,164 (23.9%) | 353 (20.3%) |  | | |
|  | 55–64 | 4,163 (17.1%) | 571 (13.7%) | 1,417 (15.2%) | 1,816 (20.0%) | 359 (20.7%) |  | | |
| **Gender, n (%)** | Male | 11,018 (45.4%) | 1,596 (38.2%) | 4,056 (43.5%) | 4,381 (48.4%) | 985 (56.7%) | **<0.001 ^a^** | | |
| **Marital status, n (%)** | Married/living with partner | 13,661 (56.2%) | 2,064 (49.5%) | 5,133 (55.0%) | 5,409 (59.7%) | 1,055 (60.8%) | **<0.001 ^a^** | | |
| **Education, n (%)** | Less than college/ university educated | 12,639 (52.0%) | 2,234 (53.5%) | 4,830 (51.8%) | 4,678 (51.6%) | 888 (51.2%) | 0.068 | | |
| **Employment status, n (%)** | Currently employed full time | 12,891 (53.1%) | 1,966 (47.1%) | 4,804 (51.5%) | 5,120 (56.5%) | 1,001 (57.7%) | **<0.001 ^a^** | | |
|  | Currently employed part time | 2,963 (12.2%) | 550 (13.2%) | 1,174 (12.6%) | 1,071 (11.8%) | 168 (9.7%) |  | | |
|  | Currently self-employed | 1,799 (7.4%) | 261 (6.3%) | 681 (7.3%) | 703 (7.8%) | 154 (8.9%) |  | | |
|  | Currently not employed | 5,694 (23.4%) | 1,247 (29.9%) | 2,356 (25.3%) | 1,758 (19.4%) | 333 (19.2%) |  | | |
|  | Retired | 948 (3.9%) | 149 (3.6%) | 312 (3.3%) | 407 (4.5%) | 80 (4.6%) |  | | |
| **Household income  (€ or £%), n (%)** | <20K | 5,702 (23.5%) | 1,244 (29.8%) | 2,286 (24.5%) | 1,760 (19.4%) | 412 (23.7%) | **<0.001 ^a^** | | |
|  | 20K to <50K | 11,376 (46.8%) | 1,894 (45.4%) | 4,463 (47.9%) | 4,287 (47.3%) | 732 (42.2%) |  | | |
|  | 50K to <75K | 2,887 (11.9%) | 372 (8.9%) | 1,049 (11.2%) | 1,233 (13.6%) | 233 (13.4%) |  | | |
|  | 75K to <100K | 1,106 (4.6%) | 121 (2.9%) | 352 (3.8%) | 535 (5.9%) | 98 (5.6%) |  | | |
|  | 100K to <150K | 429 (1.8%) | 58 (1.4%) | 126 (1.4%) | 197 (2.2%) | 48 (2.8%) |  | | |
|  | 150K or more | 194 (0.8%) | 20 (0.5%) | 56 (0.6%) | 79 (0.9%) | 39 (2.2%) |  | | |
| **Smoking status, n (%)** | Current Smoker | 5,406 (22.3%) | 971 (23.3%) | 2,068 (22.2%) | 1,984 (21.9%) | 383 (22.1%) | **<0.001 ^a^** | | |
|  | Former Smoker | 5,438 (22.4%) | 953 (22.8%) | 2,152 (23.1%) | 2,041 (22.5%) | 292 (16.8%) |  | | |
|  | Never Smoked | 13,451 (55.4%) | 2,249 (53.9%) | 5,107 (54.8%) | 5,034 (55.6%) | 1,061 (61.1%) |  | | |
| **Alcohol use,  n (%)** | Daily | 1,004 (4.1%) | 195 (4.7%) | 408 (4.4%) | 327 (3.6%) | 74 (4.3%) | **<0.001 ^a^** | | |
|  | 4-6 times a week | 1,300 (5.4%) | 228 (5.5%) | 486 (5.2%) | 489 (5.4%) | 97 (5.6%) |  | | |
|  | 2-3 times a week | 4,207 (17.3%) | 653 (15.6%) | 1,639 (17.6%) | 1,670 (18.4%) | 245 (14.1%) |  | | |
|  | Once a week | 4,165 (17.1%) | 591 (14.2%) | 1,585 (17.0%) | 1,689 (18.6%) | 300 (17.3%) |  | | |
|  | 2-3 times a month | 3,474 (14.3%) | 538 (12.9%) | 1,386 (14.9%) | 1,342 (14.8%) | 208 (12.0%) |  | | |
|  | Once a month or less often | 4,386 (18.1%) | 850 (20.4%) | 1,671 (17.9%) | 1,617 (17.8%) | 248 (14.3%) |  | | |
|  | Abstains | 5,759 (23.7%) | 1,118 (26.8%) | 2,152 (23.1%) | 1,925 (21.2%) | 564 (32.5%) |  | | |
| **Exercise in past month,  n (%)** | 14 days or more | 5,088 (20.9%) | 588 (14.1%) | 1,624 (17.4%) | 2,340 (25.8%) | 536 (30.9%) | **<0.001 ^a^** | | |
|  | 7 – 13 days | 4,455 (18.3%) | 554 (13.3%) | 1,674 (17.9%) | 1,889 (20.9%) | 338 (19.5%) |  | | |
|  | 4 – 6 days | 3,809 (15.7%) | 554 (13.3%) | 1,556 (16.7%) | 1,460 (16.1%) | 239 (13.8%) |  | | |
|  | 1 – 3 days | 3,332 (13.7%) | 645 (15.5%) | 1,404 (15.1%) | 1,080 (11.9%) | 203 (11.7%) |  | | |
|  | Did not exercise | 7,611 (31.3%) | 1,832 (43.9%) | 3,069 (32.9%) | 2,290 (25.3%) | 420 (24.2%) |  | | |
| **Body Mass Index, n (%)** | <18.5 kg/m^2^ | 1,085 (4.5%) | 221 (5.3%) | 433 (4.6%) | 354 (3.9%) | 77 (4.4%) | **<0.001 ^a^** | | |
|  | 18.5 to 24.9 kg/m^2^ | 11,814 (48.6%) | 1,817 (43.5%) | 4,495 (48.2%) | 4,623 (51.0%) | 879 (50.6%) |  | | |
|  | 25.0 to 29.9 kg/m^2^ | 6,546 (26.9%) | 1,039 (24.9%) | 2,522 (27.0%) | 2,526 (27.9%) | 459 (26.4%) |  | | |
|  | ≥30 kg/m^2^ | 2,836 (11.7%) | 643 (15.4%) | 1,142 (12.2%) | 903 (10.0%) | 148 (8.5%) |  | | |
|  |  | | | | | | | | |

BMI, body mass index; SD, standard deviation.

For BMI: <18.5 kg/m^2^, underweight; 18.5–24.9 kg/m^2^, normal weight; 25.0–29.9 kg/m^2^, overweight; ≥30 kg/m^2^, obese.

^a^The ANOVA F statistic or the Chi-square statistic is significant at the .05 level.
