# Supplementary material for: Profile and quality of life of the adult population in good health according to the level of vitality: European NHWS cross sectional analysis
Source: BMC Public Health. 2023 Jun 5;23:1061. doi: 10.1186/s12889-023-15754-0 (PMC10239722; doi:10.1186/s12889-023-15754-0)
Supplement: Supplementary file 3 — Additional file 3. Medical conditions and comorbidities: Total healthy population and according to vitality scores. [file 12889_2023_15754_MOESM3_ESM.docx]

Additional file 3. Medical conditions and comorbidities: Total healthy population and according to vitality scores

| **Characteristics** | **Total population (N=24,295)** | **Vitality score** | | | | | | | **P value** |
| --- | --- | --- | --- | --- | --- | --- | --- | --- | --- |
|  |  | **< 40  (N=4,173)** | **40–<50 (N=9,327)** | **50–<60 (N=9,059)** | | **≥60  (N=1,736)** | | |  |
| **Number of comorbidities (mean (SD))** | 1.3 (1.8) | 1.8 (2.2) | 1.3 (1.8) | 1.1 (1.5) | | 0.6 (1.2) | | **<0.001** | |
| **Mental disorders** |  |  |  |  | |  | | **<0.001** | |
| Anxiety^a^, n (%) | 1,494 (6.1%) | 493 (11.8%) | 627 (6.7%) | 340 (3.8%) | | 34 (2.0%) | | **<0.001** | |
| Depression^b^, n (%) | 1,790 (7.4%) | 656 (15.7%) | 715 (7.7%) | 384 (4.2%) | | 35 (2.0%) | | **<0.001** | |
| Other^c^ (no current use of medications), n (%) | 800 (3.3%) | 277 (6.6%) | 331 (3.5%) | 173 (1.9%) | | 19 (1.1%) | | **<0.001** | |
| **Pain disorders** |  |  |  |  | |  | |  | |
| Headache (no current use of medications), n (%) | 1,097 (4.5%) | 261 (6.3%) | 430 (4.6%) | 358 (4.0%) | | 48 (2.8%) | | **<0.001** | |
| Migraine (no current use of medications), n (%) | 944 (3.9%) | 242 (5.8%) | 395 (4.2%) | 276 (3.0%) | | 31 (1.8%) | | **<0.001** | |
| Pain (no current use of medications or not severe when taking medications), n (%) | 1,874 (7.7%) | 501 (12.0%) | 766 (8.2%) | 554 (6.1%) | | 53 (3.1%) | | **<0.001** | |
| **Sleep disorders** |  |  |  |  | |  | |  | |
| Insomnia (no current use of medications or not moderate to severe when taking medications), n (%) | 582 (2.4%) | 193 (4.6%) | 218 (2.3%) | 146 (1.6%) | | 25 (1.4%) | | **<0.001** | |
| Other^d^ (no current use of medications), n (%) | 624 (2.6%) | 193 (4.6%) | 233 (2.5%) | 172 (1.9%) | | 26 (1.5%) | | **<0.001** | |
| **Digestive disorders** |  |  |  |  | |  | |  | |
| GERD / acid reflux, n (%) | 878 (3.6%) | 206 (4.9%) | 397 (4.3%) | 249 (2.7%) | | 26 (1.5%) | | **<0.001** | |
| Heartburn, n (%) | 1,114 (4.6%) | 258 (6.2%) | 453 (4.9%) | 361 (4.0%) | | 42 (2.4%) | | **<0.001** | |
| Other^e^, n (%) | 547 (2.3%) | 143 (3.4%) | 243 (2.6%) | 148 (1.6%) | | 13 (0.7%) | | **<0.001** | |
| **Skin/nail disorder** |  |  |  |  | |  | |  | |
| Acne, n (%) | 750 (3.1%) | 202 (4.8%) | 307 (3.3%) | 201 (2.2%) | | 40 (2.3%) | | **<0.001** | |
| Dermatitis (no current use of medications or not moderate to severe when taking medications), n (%) | 502 (2.1%) | 92 (2.2%) | 229 (2.5%) | | 168 (1.9%) | | 13 (0.7%) | | **<0.001** |
| Eczema (no current use of medications or not moderate to severe when taking medications), n (%) | 1,120 (4.6%) | 291 (7.0%) | 448 (4.8%) | | 348 (3.8%) | | 33 (1.9%) | | **<0.001** |
| Other^f^, n (%) | 1,414 (5.8%) | 282 (6.8%) | 590 (6.3%) | | 478 (5.3%) | | 64 (3.7%) | | **<0.001** |
| **Heart/blood disorder** |  |  |  | |  | |  | |  |
| T2D^g^, n (%) | 273 (1.1%) | 59 (1.4%) | 95 (1.0%) | | 110 (1.2%) | | 9 (0.5%) | | 0.015 |
| High blood pressure (not associated with T2D or high cholesterol if current use of medications for these conditions), n (%) | 1,459 (6.0%) | 303 (7.3%) | 546 (5.9%) | | 549 (6.1%) | | 61 (3.5%) | | **<0.001** |
| High cholesterol (not associated with T2D or high blood pressure if current use of medications for these conditions), n (%) | 1,318 (5.4%) | 206 (4.9%) | 566 (6.1%) | | 492 (5.4%) | | 54 (3.1%) | | **<0.001** |
| **Respiratory disorder** |  |  |  | |  | |  | |  |
| Allergies (no current use of medications), n (%) | 2,193 (9.0%) | 414 (9.9%) | 872 (9.3%) | | 821 (9.1%) | | 86 (5.0%) | | **<0.001** |
| Asthma (no current use of medications), n (%) | 693 (2.9%) | 149 (3.6%) | 295 (3.2%) | | 226 (2.5%) | | 23 (1.3%) | | **<0.001** |
| Hay fever, n (%) | 1,835 (7.6%) | 359 (8.6%) | 672 (7.2%) | | 723 (8.0%) | | 81 (4.7%) | | **<0.001** |

GERD, gastroesophageal reflux disease; SD, standard deviation; T2D, type 2 diabetes.

^a^No current use of medications

^b^No current use of medications or not severe when taking medications

^c^Attention deficit disorder, attention deficit hyperactivity disorder, bipolar disorder, generalized anxiety disorder, obsessive compulsive disorder, panic disorder, phobias, post-traumatic disorder, social anxiety disorder (no current use of medications for all disorders)

^d^Narcolepsy (no current use of medications), sleep apnea (not severe when taking medications), other sleep difficulties (no current use of medications)

^e^Chronic constipation, diarrhea (frequent), diverticulitis, ulcers (active/peptic stomach or duodenal, no current use of medications)

^f^Atopic dermatitis (not moderate to severe when taking medications), Fungal infections of the skin or Athlete’s foot, hidradenitis suppurativa, rosacea, shingles, skin ulcers/cellulitis

^g^Not associated with high blood pressure or high cholesterol if current use of medications for these conditions

The Chi-square statistic is significant at the 0.05 level.
