# Supplementary material for: Profile and quality of life of the adult population in good health according to the level of vitality: European NHWS cross sectional analysis
Source: BMC Public Health. 2023 Jun 5;23:1061. doi: 10.1186/s12889-023-15754-0 (PMC10239722; doi:10.1186/s12889-023-15754-0)
Supplement: Supplementary file 4 — Additional file 4. Descriptive statistics of the EQ-5D-5L according to the vitality score. [file 12889_2023_15754_MOESM4_ESM.docx]

Additional file 4. Descriptive statistics of the EQ-5D-5L according to the vitality score

| **Characteristics** | | **Total population**  **(N=24,295)** | **Vitality Score** | | | | **p-value** |
| --- | --- | --- | --- | --- | --- | --- | --- |
|  |  |  | **< 40 (N=4,173)** | **40 - < 50 (N=9,327)** | **50 - < 60 (N=9,059)** | **>60 (N=1,736)** |  |
| **EQ-5D-5L Index** | Mean (SD) | 0.89 (0.13) | 0.81 (0.15) | 0.87 (0.12) | 0.92 (0.11) | 0.95 (0.12) | <0.001 |
|  | Median | 0.88 | 0.82 | 0.86 | 1.00 | 1.00 |  |
|  | Q1 - Q3 | 0.82- 1.00 | 0.77- 0.88 | 0.82- 1.00 | 0.86- 1.00 | 1.00- 1.00 |  |
|  | min - max | 0.11- 1.00 | 0.11- 1.00 | 0.11- 1.00 | 0.11- 1.00 | 0.11- 1.00 |  |
| **EQ-5D-5L VAS** | Mean (SD) | 79.99 (18.57) | 68.58 (21.30) | 77.75 (17.58) | 85.60 (15.30) | 90.17 (15.76) | <0.001 |
|  | Median | 85.00 | 71.00 | 80.00 | 90.00 | 95.00 |  |
|  | Q1 - Q3 | 74.00- 91.00 | 60.00 -84.00 | 70.00- 90.00 | 80.00 -95.00 | 90.00- 100.00 |  |
|  | min - max | 0.00- 100.00 | 0.00- 100.00 | 0.00- 100.00 | 0.00- 100.00 | 0.00- 100.00 |  |
| **Mobility, n (%)** | No problems | 20740 (85.4%) | 3170 (76.0%) | 7766 (83.3%) | 8190 (90.4%) | 1614 (93.0%) | <0.001*a* |
|  | Slight problems | 2471 (10.2%) | 646 (15.5%) | 1095 (11.7%) | 650 (7.2%) | 80 (4.6%) |  |
|  | Moderate problems | 822 (3.4%) | 247 (5.9%) | 386 (4.1%) | 166 (1.8%) | 23 (1.3%) |  |
|  | Severe problems | 163 (0.7%) | 76 (1.8%) | 50 (0.5%) | 30 (0.3%) | 7 (0.4%) |  |
|  | Extreme problems | 99 (0.4% | 34 (0.8%) | 30 (.3%) | 23 (0.3%) | 12 (0.7%) |  |
| **Self-Care, n (%)** | No problems | 22924 (94.4%) | 3808 (91.3%) | 8735 (93.7%) | 8726 (96.3%) | 1655 (95.3%) | <0.001*a* |
|  | Slight problems | 789 (3.2%) | 231 (5.5%) | 337 (3.6%) | 185 (2.0%) | 36 (2.1%) |  |
|  | Moderate problems | 421 (1.7%) | 95 (2.3%) | 211 (2.3%) | 96 (1.1%) | 19 (1.1%) |  |
|  | Severe problems | 88 (0.4%) | 21 (0.5%) | 22 (0.2%) | 31 (0.3%) | 14 (0.8%) |  |
|  | Extreme problems | 73 (0.3%) | 18 (0.4%) | 22 (0.2%) | 21 (0.2%) | 12 (0.7%) |  |
| **Usual Activities, n (%)** | No problems | 20672 (85.1%) | 2875 (68.9%) | 7785 (83.5%) | 8376 (92.5%) | 1636 (94.2%) | <0.001*a* |
|  | Slight problems | 2592 (10.7%) | 880 (21.1%) | 1141 (12.2%) | 517 (5.7%) | 54 (3.1%) |  |
|  | Moderate problems | 744 (3.1%) | 284 (6.8%) | 318 (3.4%) | 111 (1.2%) | 31 (1.8%) |  |
|  | Severe problems | 208 (0.9%) | 96 (2.3%) | 63 (0.7%) | 41 (0.5%) | 8 (0.5%) |  |
|  | Extreme problems | 79 (0.3%) | 38 (0.9%) | 20 (0.2%) | 14 (0.2%) | 7 (.4%) |  |
| **Pain/ Discomfort, n (%)** | No problems | 14416 (59.3%) | 1822 (43.7%) | 4932 (52.9%) | 6195 (68.4%) | 1467 (84.5%) | <0.001*a* |
|  | Slight problems | 7736 (31.8%) | 1596 (38.2%) | 3519 (37.7%) | 2424 (26.8%) | 197 (11.3%) |  |
|  | Moderate problems | 1764 (7.3%) | 592 (14.2%) | 759 (8.1%) | 363 (4.0%) | 50 (2.9%) |  |
|  | Severe problems | 315 (1.3%) | 139 (3.3%) | 101 (1.1%) | 61 (0.7%) | 14 (0.8%) |  |
|  | Extreme problems | 64 (0.3%) | 24 (0.6%) | 16 (0.2%) | 16 (0.2%) | 8 (0.5%) |  |
| **Anxiety/ Depression, n(%)** | No problems | 15145 (62.3%) | 1450 (34.7%) | 5103 (54.7%) | 7050 (77.8%) | 1542 (88.8%) | <0.001*a* |
|  | Slight problems | 6072 (25.0%) | 1381 (33.1%) | 2977 (31.9%) | 1587 (17.5%) | 127 (7.3%) |  |
|  | Moderate problems | 2265 (9.3%) | 891 (21.4%) | 994 (10.7%) | 336 (3.7%) | 44 (2.5%) |  |
|  | Severe problems | 612 (2.5%) | 322 (7.7%) | 212 (2.3%) | 65 (0.7%) | 13 (0.7%) |  |
|  | Extreme problems | 201 (0.8%) | 129 (3.1%) | 41 (0.4%) | 21 (0.2%) | 10 (0.6%) |  |

EQ-5D-5L, EuroQol 5-Dimension Health; SD, standard deviation.

^a^The Chi-square statistic is significant at the 0.05 level.
